# The association of *N*-palmitoylethanolamine with the FAAH inhibitor URB597 impairs melanoma growth through a supra-additive action

**DOI:** 10.1186/1471-2407-12-92

**Published:** 2012-03-19

**Authors:** Laurie Hamtiaux, Julien Masquelier, Giulio G Muccioli, Caroline Bouzin, Olivier Feron, Bernard Gallez, Didier M Lambert

**Affiliations:** 1 Medicinal Chemistry, Cannabinoid and Endocannabinoid Research Group, Louvain Drug Research Institute, Université catholique de Louvain, Brussels, Belgium; 2 Bioanalysis and Pharmacology of Bioactive Lipids Laboratory, Louvain Drug Research Institute, Université catholique de Louvain, Brussels, Belgium; 3 Pole of Pharmacology and Therapeutics, Institute of Experimental and Clinical Research, Université catholique de Louvain, Brussels, Belgium; 4 Biomedical Magnetic Resonance, Louvain Drug Research Institute, Université catholique de Louvain, Brussels, Belgium

## Abstract

**Background:**

The incidence of melanoma is considerably increasing worldwide. Frequent failing of classical treatments led to development of novel therapeutic strategies aiming at managing advanced forms of this skin cancer. Additionally, the implication of the endocannabinoid system in malignancy is actively investigated.

**Methods:**

We investigated the cytotoxicity of endocannabinoids and their hydrolysis inhibitors on the murine B16 melanoma cell line using a MTT test. Enzyme and receptor expression was measured by RT-PCR and enzymatic degradation of endocannabinoids using radiolabeled substrates. Cell death was assessed by Annexin-V/Propidium iodine staining. Tumors were induced in C57BL/6 mice by s.c. flank injection of B16 melanoma cells. Mice were injected i.p. for six days with vehicle or treatment, and tumor size was measured each day and weighted at the end of the treatment. Haematoxylin-Eosin staining and TUNEL assay were performed to quantify necrosis and apoptosis in the tumor and endocannabinoid levels were quantified by HPLC-MS. Tube formation assay and CD31 immunostaining were used to evaluate the antiangiogenic effects of the treatments.

**Results:**

The *N*-arachidonoylethanolamine (anandamide, AEA), 2-arachidonoylglycerol and *N*- palmitoylethanolamine (PEA) reduced viability of B16 cells. The association of PEA with the fatty acid amide hydrolase (FAAH) inhibitor URB597 considerably reduced cell viability consequently to an inhibition of PEA hydrolysis and an increase of PEA levels. The increase of cell death observed with this combination of molecules was confirmed in vivo where only co-treatment with both PEA and URB597 led to decreased melanoma progression. The antiproliferative action of the treatment was associated with an elevation of PEA levels and larger necrotic regions in the tumor.

**Conclusions:**

This study suggests the interest of targeting the endocannabinoid system in the management of skin cancer and underlines the advantage of associating endocannabinoids with enzymatic hydrolysis inhibitors. This may contribute to the improvement of long-term palliation or cure of melanoma.

## Background

Melanoma is a malignant tumor of melanocytes with a rate of incidence considerably increasing worldwide and a poor prognosis [1]. Prevention and early detection are the most successful measures against this skin cancer. Management of advanced and metastatic melanoma currently consists of cytokine therapy and chemotherapy with drugs including Dacarbazine which is the most active single agent [2,3]. Nevertheless, frequent failing of conventional treatments led to development of novel therapeutic strategies for improvement of long-term palliation or cure of melanoma.

The implication of the endocannabinoid system in cell proliferation, differentiation and survival is now well recognized. Besides endocannabinoid levels and receptor expression varying frequently in cancer process, cannabinoids modify cell fate and decrease tumor proliferation and propagation [4]. The endocannabinoid system is constituted of the G protein-coupled cannabinoid receptors CB_1 _and CB_2_, endogenous ligands binding to the cannabinoid receptors (i.e. endocannabinoids) [5,6], as well as proteins implicated in their synthesis and degradation. *N*-arachidonoylethanolamine (AEA, anandamide) and 2-arachidonoylglycerol (2-AG) are the two major bioactive lipids activating the cannabinoids receptors [7]. Additionally, other endogenous mediators associated to the endocannabinoid system, including *N*-palmitoylethanolamine (PEA), exert their effects without binding to the CB_1 _and CB_2 _cannabinoid receptors. Indeed, many studies indicate that cannabinoids can also regulate cell functions independently of CB_1 _and CB_2 _cannabinoid receptors. Apart from binding to cannabinoid receptors, endocannabinoids can activate the vanilloid receptor 1 (TRPV1) [8], two G protein-coupled receptors - GPR55 and GPR119 [9] - as well as the peroxisome proliferator-activated receptors (PPAR's) [10]. The inactivation of endocannabinoids belonging to the *N-*acylethanolamine family - AEA and PEA - occurs essentially by enzymatic hydrolysis by the fatty acid amide hydrolase (FAAH) [11]. The *N-*acylethanolamine-hydrolyzing acid amidase (NAAA) also hydrolyses these endocannabinoids according to the same reaction with PEA as the preferred substrate [12]. On the other hand, 2-AG levels are for the most part regulated by the monoacylglycerol lipase (MAGL) [13,14] even though the alpha/beta-hydrolases 6 and 12 (ABHD6 and ABHD12) were also described to hydrolyse 2-AG [15,16].

Endocannabinoids were reported to induce growth arrest [17-20], to induce apoptosis and necrosis [21-23], to inhibit angiogenesis [24] and to possess antimetastatic effects [25-28]. Conversely, PEA was described to be devoid of antiproliferative properties by itself although it can act as an "entourage" agent by enhancing AEA cytostatic effects. This might be attributed to a down-regulation of FAAH expression or to a modulation of TRPV1 activity resulting in increased AEA mediated effects [29,30]. Blazquez et al. revealed the potential benefits of the cannabinoid system in the treatment of cutaneous melanoma. They showed that cannabinoid receptor agonists could decrease growth, proliferation, angiogenesis and metastasis of this malignant cancer [31].

In the present study, we further demonstrate the implication of endocannabinoids in malignancy and suggest the interesting possibility of developing antimelanoma therapies targeting the endocannabinoid system. Thus, we investigated whether increasing endocannabinoid levels, either by direct administration or by reducing their enzymatic degradation, or both, has an impact on the growth of an aggressive skin cancer cell line. By enhancing PEA levels through the inhibition of its FAAH-mediated hydrolysis and by direct administration, we put into light the possibility of potentiating the increase of B16 melanoma cell death and slowing tumor progression.

## Methods

### Drugs

*N*-palmitoylethanolamine and palmitic acid were obtained from Tocris Bioscience. The enzyme inhibitors URB597, CAY10402 and CAY10499 were bought from Cayman Europe and MAFP from Tocris Bioscience. CCP (*N*-cyclohexanecarbonylpentadecylamine) was kindly synthesized in our lab by Coco N. Kapanda (Université catholique de Louvain, Belgium) according to the synthetic procedure described by Vandevoorde [32] and Tsuboi [33]. All the receptor antagonists (AM251, capsazepine, GW6471, T0070907 and (-)-cannabidiol) were purchased from Tocris Bioscience. All drugs were prepared as 20 mM stock solutions in DMSO and extemporaneously diluted in media for the experiments conducted on cells. The final concentration of DMSO was kept below 0.2%. [^3^H]-anandamide (60 Ci/mmol), [^3^H]-2-oleoylglycerol (40 Ci/mmol) and [^3^H]-PEA (20 Ci/mmol) were purchased from American Radiolabeled Chemicals (St Louis, MO, USA).

### Cell culture and mouse model

The murine melanoma cell line B16 was obtained from the American Type Culture Collection and routinely cultured in Minimum Essential Medium (MEM) α medium supplemented with 10% fetal bovine serum, 100 UI/ml penicillin, 100 mg/ml streptomycin and MEM Vitamins Solution. The human melanoma cell line MZ2-MEL.43 was kindly given by Pierre Coulie (Université catholique de Louvain, Belgium) and cultured in Iscove's medium supplemented with 10% fetal calf serum. Cells were maintained at 37°C in a humidified atmosphere of 5% CO_2_.

Tumors were induced in 5 week-old male C57BL/6 mice (Elevage Janvier, France) by s.c. flank injection of 10^6 ^B16 melanoma cells. When the tumor reached a volume of 20-40 mm^3^, mice were randomly divided in groups and injected i.p. for six days with vehicle or treatment (either PEA and/or URB597 at 10 mg/kg/day, daily). Tumor size was calculated according to the following formula: (4π/3) × (width/2)^2 ^× (length/2). The procedure was approved by a local ethical review committee according to national animal care regulations.

### Enzymatic activity and inhibition

#### On cell homogenates

In order to detect a hydrolytic activity for *N*-acylethanolamines (AEA and PEA) or 2-monoacylglycerols (2-oleoylglycerol, 2-OG) in B16 cells, radiolabeled substrates - either [^3^H]-anandamide, [^3^H]-2-oleoylglycerol or [^3^H]-N-palmitoylethanolamine (25 μl, 50000 dpm, 1 nM) - were incubated in glass tubes for 10 min at 37°C with increasing amounts of cell homogenates (160 μl, 10 mM Tris-HCl, 1 mM EDTA, pH 7.4) and 10 μl of DMSO. Reactions were stopped by rapidly placing the tubes in ice-cold water, followed by the addition of cold chloroform-methanol (1:1 v/v, 400 μl). After centrifugation (850 *g*, 5 min, 4°C), the radioactivity in the aqueous phase (200 μl) was counted by liquid scintillation (UltimaGold from Perkin-Elmer). To estimate the inhibition potential on B16 cell homogenates of the inhibitors, a set amount of homogenate was chosen (25 μg of protein/tube) and compounds in DMSO (10 μl), or DMSO alone for control, were added. As control for chemical hydrolysis, dpm values obtained for tubes containing buffer instead of proteins were systematically subtracted.

#### On living cells

Cells were seeded 24 h before treatment at a concentration of 10^5 ^cells/well in a 24-well plate. The medium was removed and replaced by 200 μl of fresh medium 30 min before the beginning of the experiment. Test compounds were added to each well (150 μl) followed by the radiolabeled substrate (50 μl, 50000 dpm, 1 μM) and the plate was incubated 10 min at 37°C in a 5% CO_2 _humidified atmosphere. The reaction was stopped by adding 400 μl of cold methanol on ice. After scraping the wells, a volume of 600 μl was removed and placed in a glass tube where 300 μl chloroform were added. The tubes were centrifuged (850 *g*, 10 min, 4°C) and a 400 μl aliquot of the aqueous upper phase was used to measure the radioactivity by liquid scintillation (UltimaGold from Perkin-Elmer). Cells incubated with vehicle (DMSO) were used as control and wells containing no cells were used as blank.

### Reverse transcriptase-polymerase chain reaction

Total RNA was extracted from the cultured cells with the TriPure Isolation reagent (Roche). To measure mRNA expression, reverse transcription was performed using the Reverse Transcription System (Promega) and the generated cDNA was amplified by PCR using the primers mentioned in the Table 1. Polymerase chain reactions were performed according to the following parameters: 95°C for 10 min, 95°C for 3 s, 60°C for 26 s, and 72°C for 10s (45 cycles). After amplification, agarose gel electrophoresis was used to detect the expression of the genes.

Quantitative PCR (qPCR) was performed to study the quantitative mRNA expression of the FAAH and the NAAA. RPL19 was used as house keeping gene. The samples were run in a 96-well reaction plate and data were analyzed according to the 2^-ΔCT^ method.

**Table 1:** Primer sequences used for PCR amplification

RPL19	F: gaaggtcaaagggaatgtgttca	FAAH	F: gagatgtatcgccagtccgt
			
	R: ccttgtctgccttcagcttgt		R: acaggcaggcctataccctt
MAGL	F: atggtcctgatttcacctctggt	NAAA	F: ggttttatccctgtttcctgtttat
			
	R: tcaacctccgacttgttccgagaca		R: tttttgacaatacatcaccttcagct

CB_1_	F: ctgatgttctggatcggagtc	CB_2_	F: tgacaaatgacacccagtcttct
			
	R: tctgaggtgtgaatgatgatgc		R: actgctcaggatcatgtactcctt

GPR55	F: atttggagcagaggcacgaacatga	TRPV1	F: aactcttacaacagcctgtattccaca
			
	R: agtggcgatatagtccagcttcct		R: aagacagccttgaagtcatagttct

PPARα	F: caacggcgtcgaagacaaa	PPARγ	F: ctgctcaagtatggtgtccatga
			
	R: tgacggtctccacggacat		R: tgagatgaggactccatctttattca

### MTT cell viability assay

The effect on cell viability of the different treatments was measured using MTT assay, which is based on the transformation of 3-(4,5-dimethylthiazol-2-yl)-2,5-diphenyltetrazolium bromide (MTT) in formazan crystals by the mitochondrial succinate dehydrogenase of viable cells. Cells were plated in 96-well plates at a density of 2000 cells/well in medium supplemented with 10% serum. After 5 h of incubation at 37°C in a 5% CO_2 _humidified atmosphere, test compounds diluted in culture medium were added in each well for 24 h, 48 h or 72 h. The medium was then removed and 100 μl of MTT solution (0.3 mg/ml) were added for a 2 h incubation. The MTT solution was removed, replaced by 100 μl DMSO to dissolve the crystalline formazan product and the absorbance was read at 570 nm (with a reading at 650 nm as reference) using a microplate spectrophotometer.

For the treatments with the receptor antagonists, only the 72 h time point was considered and the antagonists were added 1 h before the beginning of the cytotoxic treatment.

### Annexin-V/propidium iodide staining

Detection and quantification of apoptosis was performed by the analysis of phosphatidylserine on the outer leaflet of apoptotic cell membranes using Annexin-V-Fluorescein. Propidium iodide was used for the differentiation from necrotic cells. Cells were incubated for 24 h with the cytotoxic treatment before being stained with the Roche Annexin-V-FLUOS Staining kit (Mannheim, Germany) following the manufacturer's instructions. Cells were examined using a fluorescence microscope from Optika (Ponteranica, Italy). Pictures were taken with a Moticam 2300 from Motic (Hong Kong, China).

### Endocannabinoid quantification by HPLC-MS

Cells (10^7 ^cells/condition) were seeded in 10% FBS media for 12 h prior to the incubation (8 h) with drugs, or vehicle, in 1% FBS media. Cells and media were then recovered and the lipids extracted, in the presence of deuterated standards (200 pmol), by adding 14 ml of chloroform and 7 ml of methanol. Following vigorous mixing and sonication, the samples were centrifuged and the organic layer was recovered and then dried under a stream of N_2_.

Endocannabinoid levels in B16 tumors were quantified by directly homogenizing the tissue in chloroform (14 ml) before adding the deuterated standards, methanol (7 ml) and water (3.5 ml). After mixing and phase separation by centrifugation, the organic layer was recovered and dried under a stream of N_2_.

Both for cells and tumors, the resulting lipid extracts were purified by solid-phase extraction using silica and elution with an EtOAc-Acetone (1:1) solution [34,35]. The resulting lipid fraction was analysed by HPLC-MS using an LTQ Orbitrap mass spectrometer (ThermoFisher Scientific) coupled to an Accela HPLC system (ThermoFisher Scientific) [36]. Analyte separation was achieved using a C-18 Supelguard pre-column and a Supelcosil LC-18 column (3 μM, 4.6 × 150 mm) (Sigma-Aldrich). Mobile phases A and B were composed of MeOH-H_2_O-acetic acid 75:25:0.1 (v/v/v) and MeOH-acetic acid 100:0.1 (v/v), respectively. The gradient (0.5 ml/min) was designed as follows: transition from 100% A to 100% B linearly over 15 min, followed by 10 min at 100% B and subsequent re-equilibration at 100% A. We performed MS analysis in the positive mode with an APCI ionisation source. The capillary and APCI vaporiser temperatures were set at 250°C and 400°C, respectively. *N*-acylethanolamines were quantified by isotope dilution using their respective deuterated standards with identical retention [34]. The data were normalized by cell number in the in vitro experiments and by tumor sample weight in the in vivo testing.

### Histology

Tumors were excised after five or six days of treatment with tested compounds or vehicle and either fixed in 4% paraformaldehyde or embedded in O.C.T. compound for standard paraffin sections or for cryosectioning, respectively. Tissue samples were sliced in 5 μm sections for paraffin samples and 10 μm sections for frozen samples. The paraffin sections were stained with Haematoxylin & Eosin and photographed on a Zeiss MIRAX microscope to allow a global overview of tumor necrosis. Induction of apoptosis was assessed by TUNEL assay using an in situ cell death detection kit (Roche Diagnostics, Vilvoorde, Belgium) on frozen slices. Vascularization was evaluated by immunostaining of tumor cryosections using an antibody directed against CD31 (BD Biosciences, San Jose, CA). Nuclei were also counterstained with 4,6-diamidino-2-phenylindole (DAPI). Necrosis, apoptosis and blood vessel area were quantified using Frida software and expressed as a percentage of the whole tumor area.

### Tube formation assay

The antiangiogenic effect of the different treatments was determined by seeding 15 × 10^3 ^endothelial cells (HUVEC) in 96-well plates containing Matrigel (BD Biosciences). Formation of capillary-like tubular structures was quantified by counting the number of tube intersections in each well.

### Statistical analysis

Values were expressed as mean ± SEM. Statistical analysis was performed by ANOVA or by unpaired Student's *t *test.

## Results

### Hydrolysis and cytotoxicity of AEA, 2-AG and PEA in B16 cells

**Figure 1:**
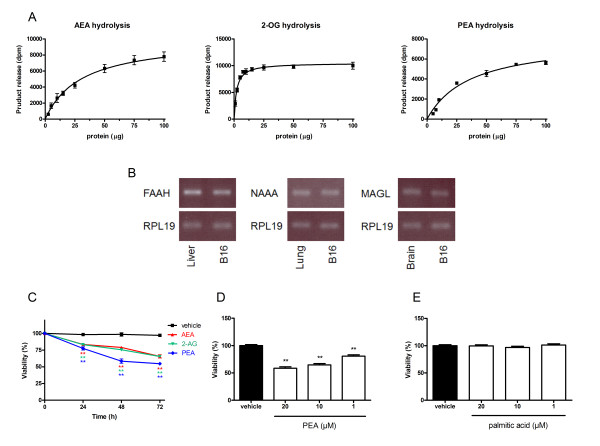
**Enzymatic hydrolysis and cytotoxicity of AEA, 2-AG and PEA in B16 melanoma cells**. (**A**) Enzymatic activities for AEA, 2-AG and PEA hydrolysis were measured in B16 cell homogenates using [^3^H]-AEA, [^3^H]-2-OG and [^3^H]-PEA, respectively. Data are the mean of three experiments performed in duplicate. (**B**) B16 cells express endocannabinoid degrading enzymes FAAH, NAAA and MAGL. Detection of mRNA was performed by RT-PCR using respectively mouse liver, lung and brain as control and RPL19 as house keeping gene (blot representative of three). (**C**) The endocannabinoids AEA, 2-AG and PEA decrease B16 cell viability. Cells were seeded 5 h before treatment (2000 cells/well in microwells) and incubated with 10 μM of endocannabinoids. After 24 h, 48 h or 72 h of treatment, cytotoxicity was assessed by a MTT test. Data are expressed as percentage of the vehicle control and are the mean of three experiments performed in quintuplicate. Significantly different (**P < 0.01) from vehicle incubation. (**D**) PEA dose-dependently decreases B16 cell viability. The cells were incubated with 1 μM, 10 μM and 20 μM of PEA. After 72 h of treatment, cytotoxicity was assessed by a MTT test. Data are expressed as percentage of the vehicle control and are the mean of three experiments performed in quintuplicate. Significantly different (**P < 0,01) from vehicle incubation. (**E**) Palmitic acid does not decrease B16 viability. The cells were incubated with 1 μM, 10 μM and 20 μM of palmitic acid. After 72 h of treatment, cytotoxicity was assessed by a MTT test. Data are expressed as percentage of the vehicle control and are the mean of three experiments performed in quintuplicate.

In order to evaluate the hydrolysis of AEA, 2-AG and PEA, we used [^3^H]-AEA, [^3^H]-2-OG and [^3^H]-PEA and found that B16 cell homogenates significantly hydrolyzed endocannabinoids (Figure 1A). When looking for the expression of enzymes responsible for endocannabinoid hydrolysis, we detected mRNA expression of the major endocannabinoid degrading enzymes, i.e. FAAH, NAAA and MAGL (Figure 1B). Since the aim of this work was to increase endocannabinoid cytotoxicity by inhibiting their hydrolysis, we ensured that AEA, 2-AG and PEA reduced cell viability in our B16 model using a MTT test. We observed that at 10 μM and already after 24 h of treatment, AEA, 2-AG and PEA decreased cell viability, in comparison to vehicle (Figure 1C). This effect was amplified after 48 h and 72 h of incubation and was slightly more pronounced for PEA. Therefore, we further investigated the cytotoxic effect of PEA and obtained a dose-response when this molecule was tested at 1, 10 and 20 μM for 72 h (Figure 1D). Because endocannabinoids' fatty acid metabolites are known to possess numerous functions we tested whether they affect cell viability of B16 cells. We found that neither palmitic acid (Figure 1E) nor arachidonic acid (data not shown) affect cell viability. Taken together these data support our hypothesis that increasing endocannabinoid levels, by blocking their degradation, would reduce tumor cell viability.

### Inhibition of *N-*palmitoylethanolamine degradation

Five inhibitors that were reported to decrease *N-*palmitoylethanolamine hydrolysis either by inhibiting FAAH (URB597, CAY10402, MAFP and CAY10499) or NAAA (CCP) (see Additional file 1) were tested at 1 μM and 10 μM on total cell homogenates. The inhibition assays were also performed on intact cells in culture to confirm that the inhibitors do reach their targets in culture conditions (Table 2).

**Table 2:** Inhibition of PEA hydrolysis in B16

		PEA hydrolysis inhibition (% ± SEM)	
		Cell homogenates	Intact cells
URB597	10 μM	***95 ± 1.0***	***93 ± 3.7***
	1 μM	***90 ± 4.1***	***85 ± 5.2***
CAY10402	10 μM	***95 ± 6.3***	***75 ± 4.6***
	1 μM	***83 ± 5.2***	***65 ± 5.1***
MAFP	10 μM	***86 ± 6.3***	***70 ± 4.2***
	1 μM	***88 ± 3.1***	***75 ± 3.0***
CAY10499	10 μM	***100 ± 7.5***	***80 ± 4.4***
	1 μM	***91 ± 3.9***	***64 ± 4.9***
CCP	10 μM	***6 ± 4.2***	***26 ± 6.8***
	1 μM	***4 ± 5.6***	***26 ± 7.0***

FAAH inhibitors (URB597, CAY10402), dual FAAH/MAGL inhibitors (MAFP, CAY10499) and NAAA inhibitor (CCP) were tested at concentrations of 1 and 10 μM on cell homogenates (25 μg protein, pH 7.4) and on living cells (10^5 ^cells/well, seeded 24 h before) in culture medium. Data are the mean of three experiments and are expressed as percentage of the control containing vehicle instead of the inhibitors

**Figure 2:**
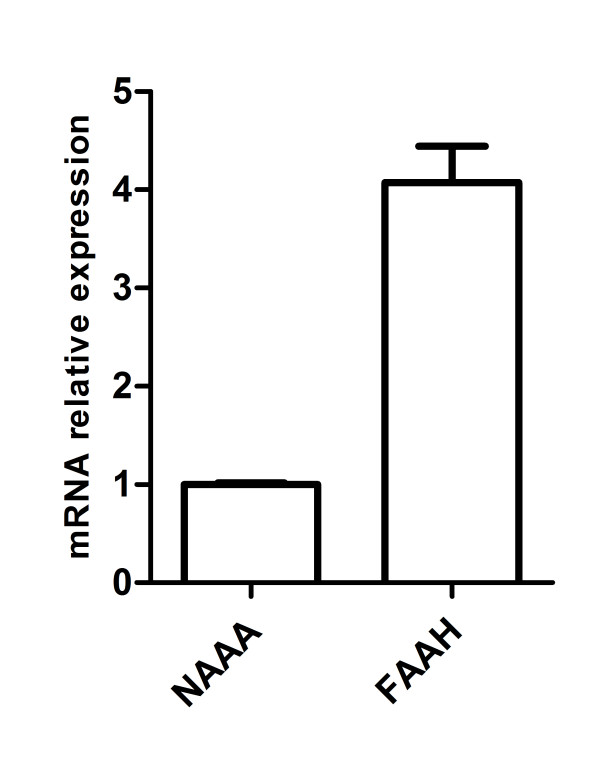
**FAAH and NAAA mRNA relative expression in B16 cells**. Quantitative detection of mRNA was performed by qPCR using RPL19 as house keeping gene. Data were analyzed according to the 2^-ΔCT ^method and the NAAA mRNA expression is set at 1 value.

We observed that URB597, CAY10402, MAFP and CAY10499 all inhibit PEA hydrolysis in homogenates and cultured cells even though the inhibition is slightly less pronounced in the latter case. Note that the use of CCP did not reduce PEA hydrolysis in homogenates and only decrease it by 26% ± 7.0 in intact cells at 10 μM. The absence of inhibition observed in homogenates compared to cells in culture could be explained by a NAAA activity known to be the highest at acidic pH while the assay was performed on homogenates at physiological pH [12]. Quantitative measurements of FAAH and NAAA mRNA expression were also performed in order to investigate the possibility that a high level of FAAH, in comparison to NAAA, could lead to the lack of efficacy of CCP on PEA hydrolysis in our system. The results indicated that the relative mRNA levels of the two enzymes were in the same order of magnitude (Figure 2) and thus the lack of NAAA expression at the mRNA levels does not account for the inefficiency of CCP.

### Increase of *N-*palmitoylethanolamine effects on B16 cell viability by hydrolysis inhibitors

**Figure 3:**
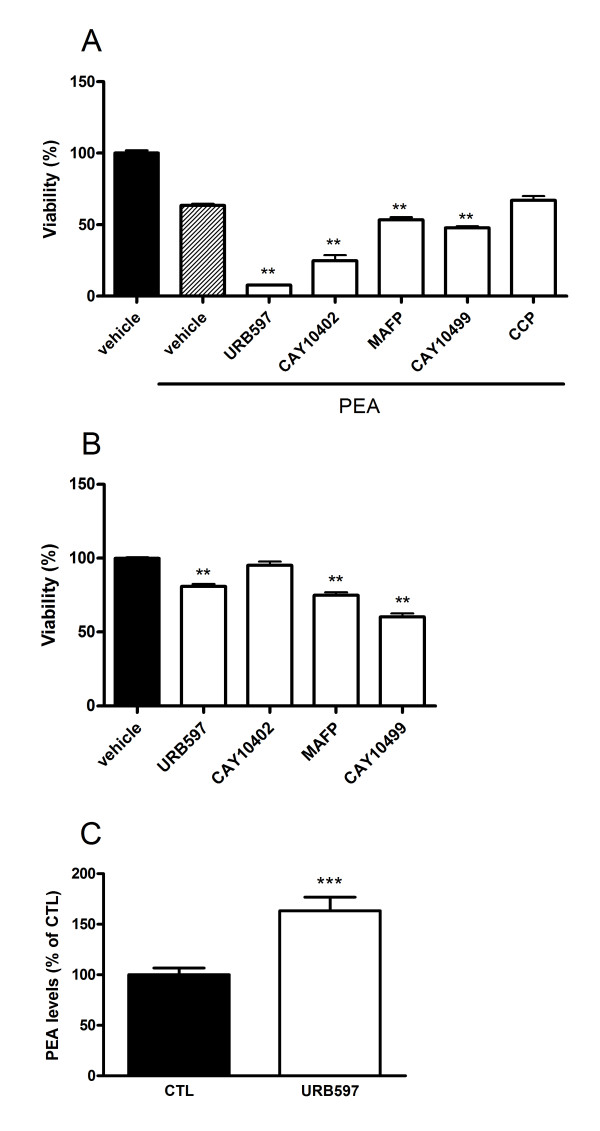
**Enhancement of PEA effects on B16 cell viability by hydrolysis inhibitors**. (A) The FAAH inhibitors (URB597, CAY10402) and dual FAAH/MAGL inhibitors (MAFP, CAY10499) potentiate PEA cytotoxicity. B16 cells were seeded 5 h before treatment (2000 cells/well in microwells) and incubated with PEA (10 μM) with or without URB597, CAY10402, MAFP, CAY10499 or CCP (at 10 μM). After 72 h of treatment, cytotoxicity was assessed by a MTT test. Data are the mean of three experiments (performed in quintuplicate) and are expressed as percentage of the vehicle control. Significantly different (**P < 0.01) from PEA incubation. (**B**) URB597, MAFP and CAY10499 slightly decrease B16 cell viability. Cells were seeded 5 h before treatment (2000 cells/well in microwells) and incubated with inhibitors at a concentration of 10 μM. After 72 h of treatment, cytotoxicity was assessed by a MTT test. Data are the mean of three experiments performed in quintuplicate and are expressed as percentage of the vehicle control. Significantly different (**P < 0.01) from vehicle incubation. (**C**) URB597 increases intracellular levels of PEA as measured by HPLC-MS. B16 cells were incubated for 8 h with URB597 (1 μM). We found in control cells 25.4 ± 3.8 pmol of PEA/10^7 ^cells. Data are the mean of three experiments performed in quadruplicate and are expressed as percentage of the vehicle control. Significantly different (***P < 0.001) from vehicle incubation.

Next, PEA (10 μM) was co-incubated for 72 h with the FAAH inhibitors - URB597, CAY10402, MAFP, CAY10499 - and the NAAA inhibitor - CCP - at 10 μM, with the objective of enhancing individual effect on cell viability (Figure 3A). All four inhibitors enhanced PEA-induced cytotoxicity although the effect was more pronounced with the selective inhibitors (URB597, CAY10402) as compared to the dual FAAH/MAGL inhibitors (MAFP, CAY10499). Of note, the co-incubation of PEA and URB597 dramatically reduced cell viability which was no more than 11% of the vehicle control after 72 h of treatment. In line with the results obtained in the enzymatic inhibition assay, CCP had no potentiating effect on cytotoxicity when added to PEA.

The effects on cell viability of the inhibitors alone were also evaluated at 10 μM and after 72 h of incubation. We did not use CCP anymore because it was poor at inhibiting PEA hydrolysis in our cellular model and did not induce supplemental cytotoxicity when co-incubated with PEA. Only the three FAAH inhibitors URB597, MAFP and CAY10499 provoked a significant increase in cytotoxicity while the reversible FAAH inhibitor CAY10402 did not influence cell viability (Figure 3B).

Because the PEA-URB597 combination produced the highest cytotoxicity (Figure 3A), we focused on these compounds for the next experiments. We wondered if URB597 could exert its cytotoxicity through inhibition of FAAH and subsequent increase of PEA concentrations in our conditions. For this purpose, PEA levels were measured in B16 cells using an isotope-dilution HPLC-MS method and we observed that incubating B16 cells with URB597 (1 μM, 8 h) resulted in increased PEA levels (163 ± 13% of the control) (Figure 3C).

### PEA and URB597 potentiate cell death of B16 melanoma cells

**Figure 4:**
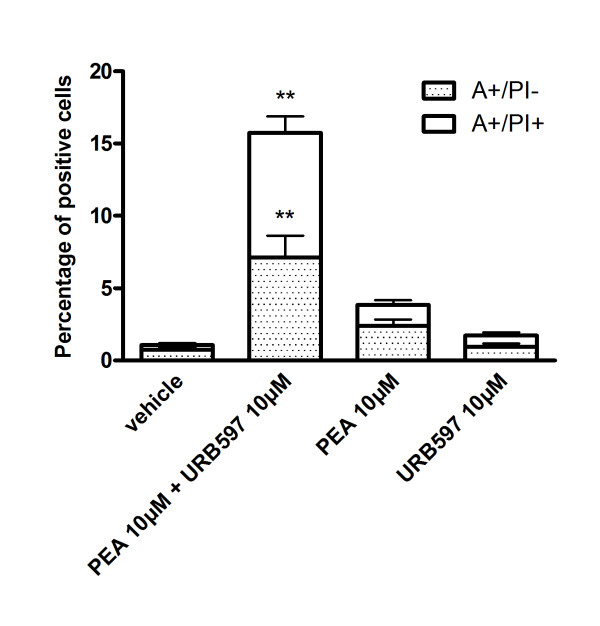
**PEA and URB597 potentiate cell death of B16 melanoma cells**. Apoptosis was assessed by Annexin-V (A)/Propidium Iodide (PI) staining. B16 cells were treated with 10 μM of PEA and/or URB597 for 24 h and the number of Annexin-V positive cells (A+/PI-, apoptotic) and of double stained cells (A+/PI+, necrotic) was expressed as a percentage of total cells. Data are the average of five random fields from experiments performed in triplicate. Significantly different (**P < 0.01) from vehicle incubation.

We next looked at the influence of PEA and URB597 on cell death by measuring annexin-V positive cells (A+/PI-) and double stained cells (A+/PI+) representing apoptotic and necrotic (or late apoptotic) cells, respectively. Translocation of phosphatidylserine is an early event in apoptosis and its measurement allows the detection of cells undergoing caspase-dependent or independent apoptosis. Treatment of B16 cells with PEA or URB597 (10 μM, 24 h) did not result in an increased number of dead cells as compared to vehicle, even though PEA tended to enhance cell death (Figure 4). On the contrary, co-incubation with PEA and URB597 increased the percentage of cells dying by both apoptosis and necrosis.

We then asked if the cytotoxic effects of the PEA-URB597 combination could be mediated by the classical molecular targets of endocannabinoids. Thus, we co-incubated PEA, URB597 or PEA-URB597 with AM251 (0.1 and 1 μM), capsazepine (0.1 and 1 μM), GW6471 (1 and 5 μM), T0070107 (1 and 5 μM) and cannabidiol (1 and 10 μM). These compounds are selective antagonists of the CB1, TRPV1, PPARα, PPARγ and GPR55 receptors respectively, the mRNA of which were found in B16 melanoma cells (see Additional file 2). None of the antagonists reduced the effect of PEA (10 μM) or URB597 (10 μM) alone or in combination (see Additional file 3). We would like to point out that antagonist concentrations were selected according to the literature and that we tested their own cytotoxicity to exclude the possibility that they could affect B16 cell viability by themselves (see Additional file 4). At 10 μM, cannabidiol has enhanced the cytotoxic effect of PEA and URB597 when each of these drugs was used alone. One explanation is that this compound is a weak agonist of the TRPV1 and was shown to activate this receptor in this range of concentrations [37].

### In vivo co-administration of PEA and URB597 reduces melanoma growth

**Figure 5:**
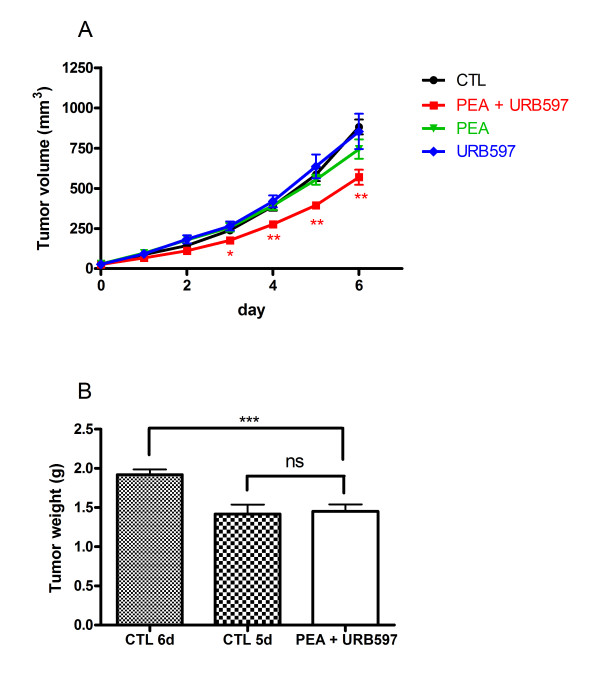
**Co-administration of PEA and URB597 reduces melanoma growth**. Tumors were induced by s.c. injection of B16 cells in C57BL/6 mice. When tumor size reached a volume of approximately 20-40 mm^2^, animals were treated i.p. either with vehicle (n = 18) or with PEA (= 7), URB597 (n = 7) or both molecules (n = 20) (at 10 mg/kg/day, daily) for six days. (**A**) Tumor volume was measured each day. (**B**) Weight of tumors treated with vehicle (6 or 5 days of treatment) or PEA + URB597 (6 days of treatment) was measured at the end of the experiment. Significantly different (*P < 0.05; **P < 0.01; ***P < 0.001) from vehicle administration.

Since PEA and URB597 were able to induce cell death when used in co-incubation in vitro, we wanted to confirm that this was also the case in vivo. Therefore, we evaluated their effect on malignant melanoma growth in C57BL/6 mice. Tumors were generated by subcutaneous injection of B16 cells, and when tumors reached a volume of approximately 20-40 mm^3^, mice were treated by intraperitoneal injections of vehicle, PEA and/or URB597 for up to six days. The tumor size of mice treated with PEA or URB597 alone were not significantly different from those injected with vehicle. However, co-administration of PEA and URB597 resulted in a significantly reduction of tumor growth and of their size at the end of the experiment (Figure 5A). We also weighted tumors at the end of the experiment and observed a significant difference between normal and PEA/URB597 treated tumors (Figure 5B).

**Figure 6:**
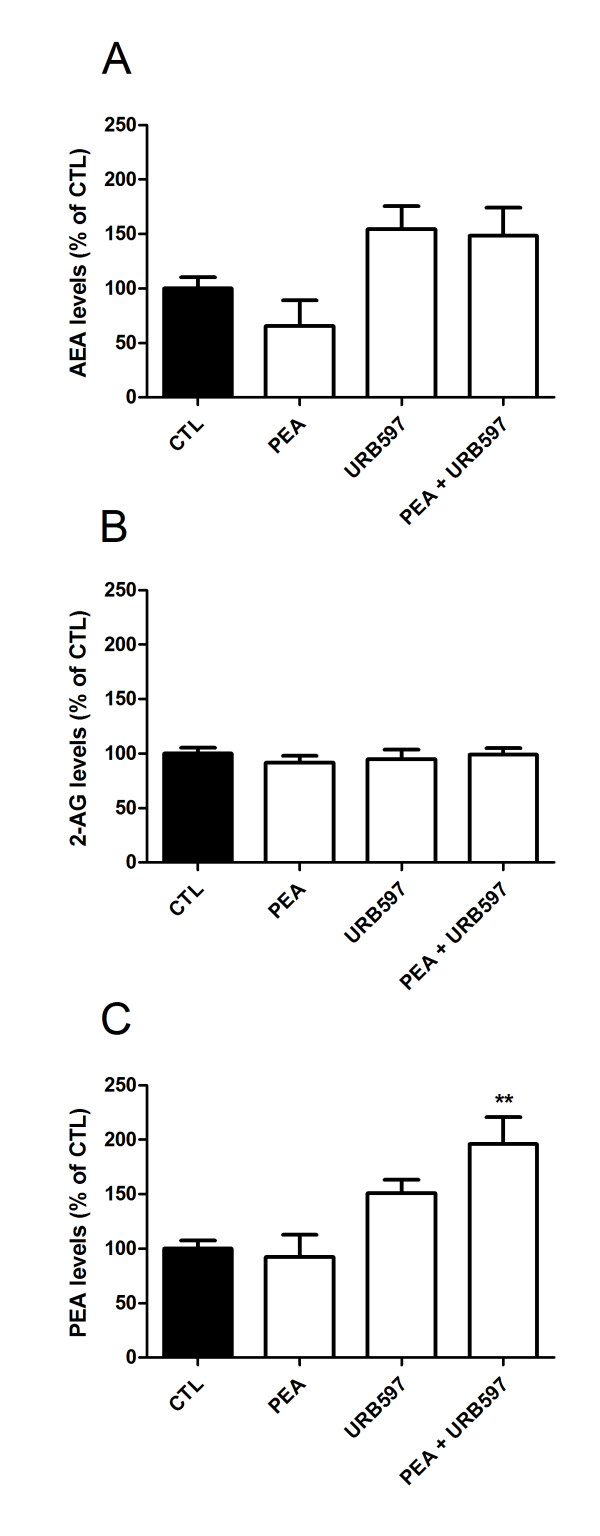
**Tumor endocannabinoid levels**. (**A**) AEA, (**B**) 2-AG and (**C**) PEA levels were measured by HPLC-MS in tumor samples of mice treated i.p. with vehicle (n = 15), PEA (n = 7), URB597 (n = 7) or both molecules co-incubation (n = 15) (at 10 mg/kg/day, daily) for six days. Basal levels are 78.4 ± 11.7 pmol/g, 1.55 ± 0.11 nmol/g and 546.1 ± 55 pmol/g of tissue for AEA, 2-AG and PEA, respectively. Significantly different (**P < 0.01) from vehicle administration.

With the aim to correlate the anticancerous effect of PEA and URB597 with endocannabinoid levels inside the tumor, we measured AEA, 2-AG and PEA amounts in the excised tumors by HPLC-MS. On the one hand, AEA and 2-AG levels were not significantly affected by PEA, URB597 or both molecules injection, even though AEA concentration tended to increase after URB597 or PEA/URB597 treatments (Figure 6A, B). On the other hand, PEA levels were increased after co-treatment with PEA and URB597 but not if these compounds were injected alone, even though a trend towards increased levels was observed following URB597 administration (Figure 6C).

### PEA and URB597 co-administration induces tumor necrosis

**Figure 7:**
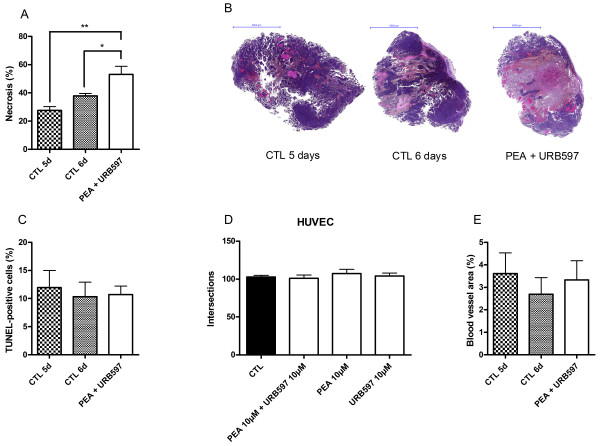
**PEA and URB597 increase tumor necrosis**. Tumors treated daily by co-administration of PEA and URB597 were excised after six days of treatment and tumors treated by vehicle were excised after five or six days. (**A**) Quantification of necrotic regions was realized using Haematoxylin and Eosin staining (n = 5). Significantly different (*P < 0.05; **P < 0.01) from vehicle administration. (**B**) Pictures show representative histological H-E stainings performed on tumor slices. (**C**) Apoptosis was evaluated by TUNEL assay (n = 6-12). (D) PEA and URB597 do not affect angiogenesis. HUVEC were seeded (10^4 ^cells/well in microwells) and incubated with PEA (10 μM) and/or URB597 (10 μM). The angiogenic potency was determined by measuring the number of tube intersections formed after 24 h plating on Matrigel (n = 2). (**E**) Tumor vascularization was evaluated by CD31 immunostaining of tumor cryosections and blood vessel area was expressed as percentage of the total area (n = 5).

Necrosis and apoptosis were quantified on tumor slices after Haematoxylin & Eosin (H&E) and TUNEL staining respectively. Tumors were excised after six days of co-treatment with PEA and URB597. Vehicle-treated tumors were excised after five or six days of injection in order to be able to compare either tumor treated during the same period of time, or tumors having the same volume at the end of the experiment. Indeed, we observed that size of six days drug-treated tumors and five days vehicle-treated tumors did not significantly differ (Figure 5A, B). It is known that more voluminous tumors may present larger necrotic regions and we wanted to exclude this artifact. Here we show that tumors co-treated with PEA and URB597 present enlarged necrotic regions (53 ± 6%) as compared with both tumors treated during five or six days with vehicle (28 ± 3% and 38 ± 2% respectively) (Figure 7A). These results were consistent with observations we made during in vitro assays and demonstrate that drug treatment delays tumor progression by provoking cell death. Figure 7B displays representative tumor slices after H&E staining. TUNEL assay on tumors slices did not show more positively stained cells in treated tumors than in control tumors (Figure 7C).

Finally, we wondered if the increase in the initial level of necrosis produced by co-injection of PEA and URB597 could be the result of vascular events. Thus we performed an endothelial tube formation assay using HUVEC. Here, PEA (10 μM) and URB597 (10 μM), incubated alone or in co-incubation, did not alter the capacity of endothelial cells to form tubes when cultured on Matrigel (Figure 7D). Additionally, evaluation of tumor vascularization by immunostaining did not reveal any significant change in blood vessel area between treated and untreated mice (Figure 7E).

### PEA and URB597 impair human melanoma viability

**Figure 8:**
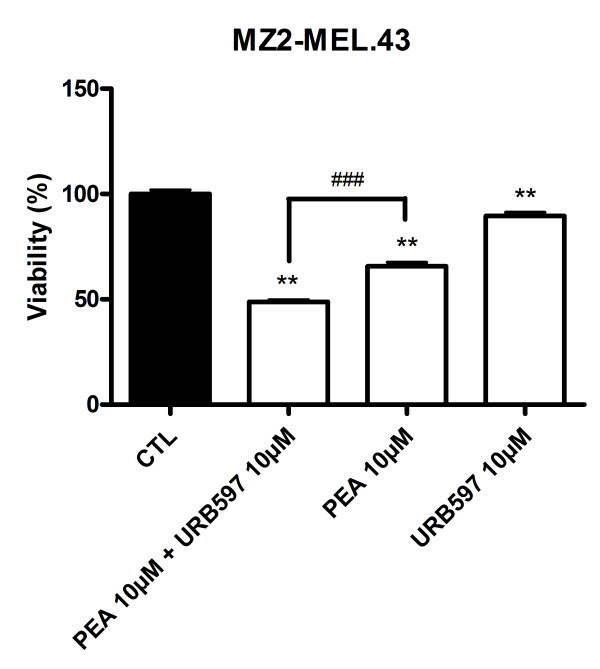
**PEA and URB597 impair human melanoma viability**. URB597 potentiates the decrease of MZ2-MEL.43 melanoma cell viability produced by PEA. Cells were seeded 5 h before treatment (2000 cells/well in microwells) and incubated with PEA (10 μM) with or without URB597 (10 μM). After 72 h of treatment, cytotoxicity was assessed by a MTT test. Data are expressed as percentage of the vehicle control and are the mean of three experiments performed in triplicate. Significantly different (**P < 0.01) from vehicle incubation. Significantly different (###P < 0.001) from PEA incubation.

In a view to strengthen the potential interest of using PEA and URB597-based treatment for melanoma growth management, we measured the effect of these compounds on a human melanoma cell line. URB597 (10 μM) slightly decreased cell viability of MZ2-MEL.43 melanoma, which was 90% of the vehicle control after 72 h of treatment. The cytotoxicity produced by PEA (10 μM) led to a reduction of cell viability to 66%, while it was potentiated by URB597 to reach 48% of residual viable cells (Figure 8).

## Discussion

The literature widely reports on the regulatory actions of the endocannabinoid system in health and disease, including cancer. Endocannabinoids and synthetic cannabinoids are essentially described as protective factors limiting cell proliferation, differentiation and survival as well as tumor development. In this study, we aimed at investigating the possibility of enhancing endocannabinoid cytotoxicity using inhibitors of their hydrolysis in a melanoma model.

After looking for the presence of enzymatic activity for AEA, 2-AG and PEA hydrolysis and elucidating which enzymes were present in our melanoma model, we showed a time-dependent effect of these three endocannabinoids on B16 cell viability. As frequently described for many cancer cell lines like colon cancer cells [17,21], glioma cells [20] breast cancer cells [19,25,27] or prostate cancer cells [28], AEA and 2-AG reduced B16 cell viability. Surprisingly, at 10 μM, we found PEA to decrease cell viability. Indeed, this endocannabinoid was reported to act as an "entourage" agent able to increase AEA antiproliferative effects but not to induce those when incubated alone, even at concentrations up to 10 μM [29,30]. However, here PEA could clearly reduce B16 cell viability at 10 μM but also at lower concentrations. We also confirmed that PEA degradation into palmitic acid was not responsible for the effects observed with PEA [38].

We then sought to increase PEA levels to investigate if this could affect B16 melanoma cell viability by potentiating PEA cytotoxicity. Some reports indicate that the use of inhibitors of endocannabinoid hydrolysis can be of interest in the development of anticancer therapies. For example, elevation of endocannabinoid concentrations by inhibitors of their re-uptake and degradation produced a decrease in thyroid transformed cells growth [39]. In a colorectal cancer cell line, inhibitors of endocannabinoid inactivation increased their levels and reduced cell proliferation [40]. Other experiments performed on prostate cancer cells also testified of the benefits of inhibiting 2-AG hydrolysis to block cell growth and invasion [28,41-43]. Therefore, we assayed five inhibitors of either FAAH or NAAA, the two main enzymes known to hydrolyze PEA and that we found to be expressed in B16 melanoma cells. The most cytotoxic treatment was obtained by the co-incubation of 10 μM of PEA with the irreversible FAAH inhibitor, URB597 at 10 μM. Using a human melanoma cell line, we also evidenced a significant cytotoxicity of this treatment. Interestingly, among the inhibitors tested, the highest inhibition of PEA hydrolysis was obtained with URB597. This compound already exerted a significant decrease in cell viability when used alone. Of note, the other selective FAAH inhibitor CAY10402 was also able to potentiate PEA cytotoxicity without inducing any decrease in cell viability by itself. This last observation suggested that the PEA-URB597 cytotoxicity might be partly due to elevation of endocannabinoid levels. Indeed we found that incubation of B16 cells with URB597 could raise PEA levels up to 163%, indicating that the cytotoxicity of this inhibitor could be partly assigned to modulation of PEA levels. Actually, even though the concentrations obtained when inhibiting FAAH were lower than those required to reduce cell viability by adding PEA exogenously, we considered that locally available PEA levels might be high enough to produce pharmacological effects when inhibiting FAAH. The higher effects, when looking at PEA hydrolysis or at cell viability, of URB597 compared to CAY10402 could be related to a reversible FAAH inhibition by CAY10402, while URB597 was characterized as an irreversible inhibitor. In the liver, URB597 was previously shown to enhance AEA-induced cell death via FAAH inhibition [44]. Surprisingly, the two dual FAAH/MAGL inhibitors MAFP and CAY10499 only slightly accentuated PEA effects on cells viability even though they were effective at inhibiting PEA hydrolysis and exhibited cytotoxic effects by themselves. Nevertheless, in comparison to URB597, the effect of MAFP on intracellular PEA levels was less pronounced and CAY10499 did not significantly affect PEA concentration even though it tended to increase it (see Additional file 5A). It is therefore supposed that the action of these two dual inhibitors is not sufficient to increase PEA levels and, consequently, its cytotoxicity upon melanoma cells. In addition, as mentioned above these compounds are also irreversible inhibitors of MAGL and consequently can influence 2-AG levels as well (see Additional file 5B). The inhibition of 2-AG degradation was frequently evidenced to result in antitumor effects, either by making profit of 2-AG antiproliferative and anti-invasive properties [40,41,43] or by limiting the production of arachidonic acid known to be associated to aggressiveness of cancer cells [45,46]. Since this endocannabinoid also exhibited cytotoxic properties in B16 cells, we would have assumed that the concomitant inhibition of the FAAH and the MAGL should have produced an enlarged diminution of cell viability. Astonishingly, we only observed that inhibition of 2-AG hydrolysis produced a small decrease in cell viability and poorly contributed to induction of cytotoxicity when combining to PEA. This suggests a minor role of 2-AG in cell viability in our model as compared to PEA. Finally, although the poor inhibition of PEA hydrolysis by the NAAA inhibitor is puzzling at a first glance, the almost full inhibition of PEA hydrolysis by URB597 suggests that FAAH is likely to account for most of PEA degradation in our cellular model. Thus, even if CCP inhibits the NAAA-mediated PEA hydrolysis, FAAH can largely compensate for the decreased NAAA activity [47].

The receptor mediating the cytotoxic effects of PEA and URB597 could not be identified as one of the classical molecular targets mediating endocannabinoid action. However, though pharmacological blockade of receptors constitutes a reliable and widely used method, silencing of these receptors may constitute a matter of interesting perspective to completely rule out their implication in the cytotoxic effects produced by the treatments.

Co-treatment of PEA and URB597 induced cell death in cultured B16 melanoma cells, while PEA and URB597 incubated alone only slightly increased the number of apoptotic and necrotic cells. This drug activity reinforcement was confirmed in vivo where tumor volume and tumor weight were decreased after 6 days of treatment only when melanoma-bearing mice were treated with both PEA and URB597. When looking at the endocannabinoid levels in the tumor after treatment, the growth delay induced by PEA-URB597 treatment appears to be related to an elevation of PEA levels within the tumor. Conversely, AEA and 2-AG levels were not significantly affected by treatments even though AEA levels tended to increase following URB597 injections. These results contrast with the observations made by Bifulco et al. with rat thyroid transformed cells, in which tumor levels of AEA, 2-AG and PEA were all three augmented after an intratumor treatment with the FAAH inhibitor arachidonoyl-serotonin [39]. This difference may arise from variations in the experimental conditions, such as the injection modalities, resulting in variable availabilities of the inhibitor, or the timing at which tumors were resected. Since our results show a tendency to increase for *N-*acylethanolamine concentrations in URB597-treated mice and since only the co-incubation of PEA and URB597 increased PEA levels, we may think that the elevation of AEA and PEA levels are transitory and that these molecules are rapidly degraded. Along this line, we support the hypothesis that an earlier excision of the tumors after the last injection could have revealed a significant increase in AEA and PEA concentrations. Nevertheless, in our melanoma system, only the co-injection of PEA and URB597 is able to sufficiently increase the concentrations in order to reduce tumor growth. In addition, some reports have shown that FAAH inhibition induces an increase in 2-AG levels [39,41]. However, in our melanoma cells, 2-AG levels were not influenced at all by URB597 treatments. This may be attributed to the fact that FAAH is weakly responsible for 2-AG hydrolysis in B16 cells.

We could also evidence that the decrease in tumor growth observed with PEA-URB597 treatment was the result of increased necrotic events in the tumor. Although tumor growth delay obtained with PEA and URB597 may look marginal, the extent of necrosis observed in this very aggressive tumor model indicates that measurements of tumor volume/weight certainly underestimate the real impact of the co-treatment. Furthermore, because neither PEA nor URB597 or the association of both molecules produced antiangiogenic effects, a reduced oxygen and nutrient supply is unlikely to account for the increased necrosis induced by the treatment. It seemed of interest to investigate this point because PEA and analogues have already been described as owning antiangiogenic effects in a model of chronic inflammation [48,49]. Likewise, AEA was reported to influence cancer growth via inhibition of angiogenesis [24] and synthetic cannabinoids WIN-55.212-2 and JWH-133 were shown to decrease melanoma vascularization [31].

A large number of reports suggest the therapeutic interest of using PEA in medicine. This lipid mediator has been emerging as a potent antinociceptive molecule [50,51] and exhibits anti-inflammatory properties [52]. Of note, PEA is already used as the active molecule of anti-inflammatory and analgesic preparations (e.g. Normast^®^, Pelvilen^®^) [53,54]. These advantageous effects associated with the present observations put into light the possibility of emerging therapies implicating PEA for pathological conditions including cancer.

## Conclusions

The current study demonstrates the potential implication of endocannabinoids in B16 melanoma cell survival. Specifically, the supra-additive action of PEA and the FAAH inhibitor URB597 promotes cell death and delays in vivo tumor growth. Additionally, we confirmed that antiangiogenic events are not responsible for the enhanced necrosis observed in the tumors. Hence, this report suggests the attractive prospect of designing PEA-based anticancer therapies, with potential anti-inflammatory and antinociceptive effects, via an inhibition of its hydrolysis.

## Abbreviations

AEA: Anandamide *N*-arachidonoylethanolamine; 2-AG: 2-arachidonoylglycerol; CB_1_: Cannabinoid receptor 1; CB_2_: Cannabinoid receptor 2; FAAH: Fatty acid amide hydrolase; MAGL: Monoacylglycerol lipase; MTT: 3-(4,5-dimethylthiazol-2-yl)-2,5diphenyltetrazolium bromide; NAAA: *N*-acylethanolamine-hydrolyzing acid amidase; PEA: *N*-palmitoylethanolamine; PPARα: Peroxisome proliferator-activated receptor alpha; PPARγ: Peroxisome proliferator-activated receptor gamma; TRPV1: Transient receptor potential cation channel subfamily V, member 1

## Competing interests

The authors declare that they have no competing interests.

## Authors' contributions

LH carried out the experimental studies, performed the statistical analysis and drafted the manuscript. JM carried out the endocannabinoid quantification by HPLC-MS. GGM participated in the design of the study and carried out the endocannabinoid quantification by HPLC-MS. CB and OF participated to the in vivo experiments. BG and DML conceived the study and participated in its design and coordination and helped to draft the manuscript. All authors read and approved the final manuscript.

## Pre-publication history

The pre-publication history for this paper can be accessed here:

http://www.biomedcentral.com/1471-2407/12/92/prepub

## Supplementary Material

Additional file 1 **Structures of the endocannabinoid metabolism inhibitors used in this study**. Click here for file

Additional file 2 **Receptor expression in B16 cells**. B16 cells express cannabinoid receptor CB_1 _but not CB_2_, G-protein coupled receptor GPR55, vanilloid receptor TRPV1 and nuclear receptors PPARα and PPARγ. Detection of mRNA was performed by RT-PCR using mouse brain, spleen and liver as control and RPL19 as house keeping gene. The blots are representative of three. Click here for file

Additional file 3 **Investigation of the potential molecular targets of PEA and URB597 in B16 cells**. Cytotoxicity of PEA (10 μM), URB597 (10 μM) and PEA + URB597 was not significantly affected by CB_1 _receptor antagonist (0.1 and 1 μM), TRPV1 receptor antagonist (0.1 and 1 μM), PPAR's receptor antagonists (1 and 5 μM) and GPR55 receptor antagonist (1 and 10 μM). B16 cells were seeded 5 h before treatment (2000 cells/well in microwells) and incubated with PEA alone (10 μM), URB597 alone (10 μM) and combinations of these two molecules. Antagonists were added 1 h prior to the addition of PEA and/or URB597. A MTT test was used to evaluate the percentage of viable cells remaining after 72 h. Data are the mean of three experiments performed in triplicate and are expressed as percentage of the vehicle control. Click here for file

Additional file 4 **Cytotoxicity of receptor antagonists**. Cytotoxicity of CB_1 _receptor antagonist (AM251), TRPV1 receptor antagonist (capsazepine), PPARα and PPARγ receptor antagonists (GW6471 and T0070907 respectively) and GPR55 receptor antagonist (cannabidiol, CBD). B16 cells were incubated with the antagonists for 72 h. A MTT test was used to evaluate the percentage of viable cells remaining after treatment. Data are expressed as percentage of the vehicle control and are the mean of three experiments performed in quintuplicate. Click here for file

Additional file 5 **Effect of MAFP, CAY10499 and URB597 incubation on PEA and 2-AG levels in B16 cells**. (**A**) MAFP, but not CAY10499, increases intracellular levels of PEA. We found in control cells 25.4 ± 3.8 pmol of PEA/10^7 ^cells. (**B**) MAFP and CAY10499, but not URB597, increase intracellular levels of 2-AG. We found in control cells 29.9 ± 4.8 pmol of 2-AG/10^7 ^cells. Levels were measured by HPLC-MS. B16 cells (10^7 ^cells) were incubated for 8 h with URB597, CAY10499 or MAFP (1 μM). Data are the mean of three experiments performed in quadruplicate and are expressed as percentage of the vehicle control. Significantly different (*P < 0.05; **P < 0.01; ***P < 0.001) from vehicle incubation. Click here for file
