# Supplementary figures and images for: The association of N-palmitoylethanolamine with the FAAH inhibitor URB597 impairs melanoma growth through a supra-additive action
Source: BMC Cancer. 2012 Mar 19;12:92. doi: 10.1186/1471-2407-12-92 (PMC3364151; doi:10.1186/1471-2407-12-92)

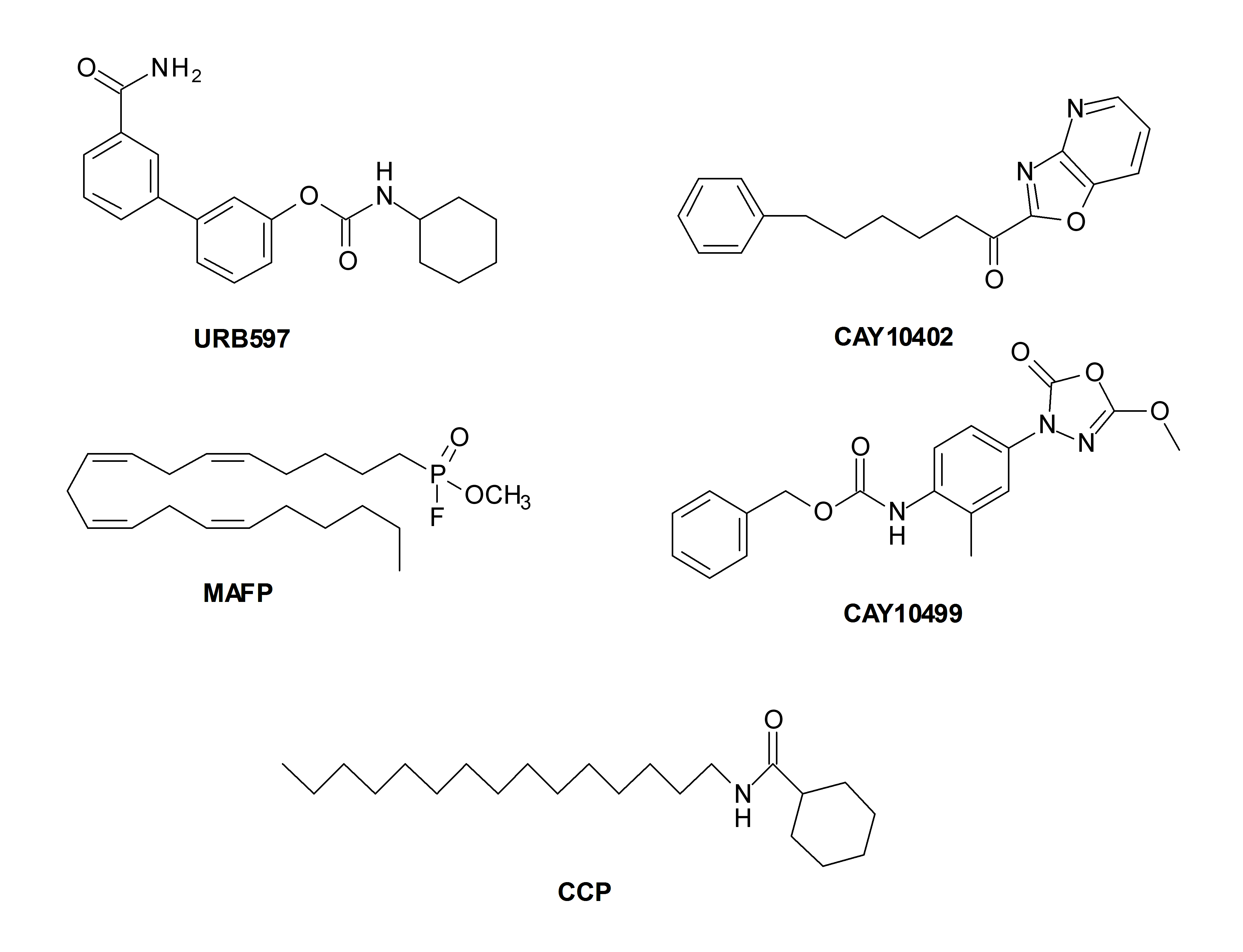

Supplement: Additional file 1 — Structures of the endocannabinoid metabolism inhibitors used in this study. [file 1471-2407-12-92-S1.TIFF]

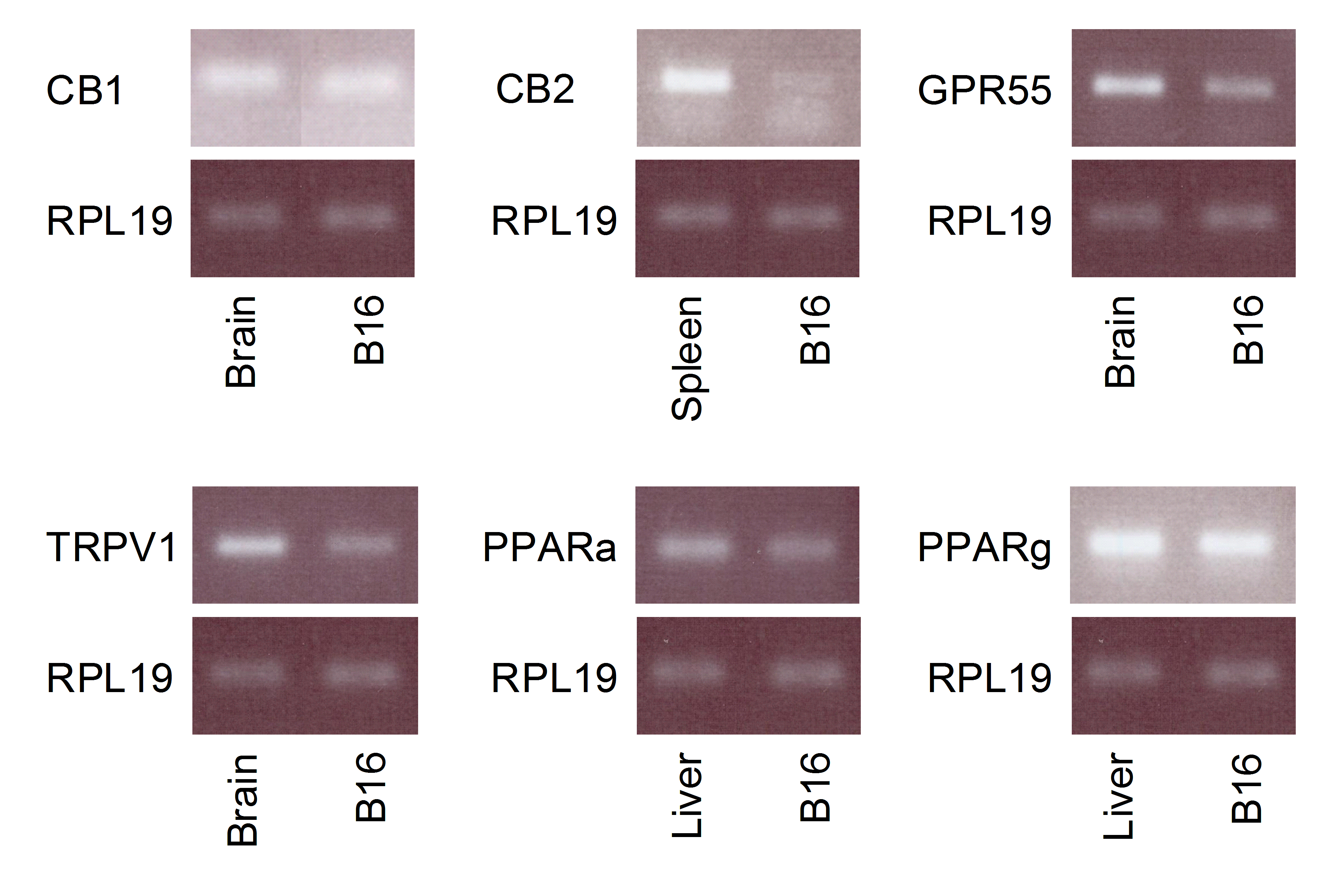

Supplement: Additional file 2 — Receptor expression in B16 cells. B16 cells express cannabinoid receptor CB1 but not CB2, G-protein coupled receptor GPR55, vanilloid receptor TRPV1 and nuclear receptors PPARα and PPARγ. Detection of mRNA was performed by RT-PCR using mouse brain, spleen and liver as control and RPL19 as house keeping gene. The blots are representative of three. [file 1471-2407-12-92-S2.TIFF]

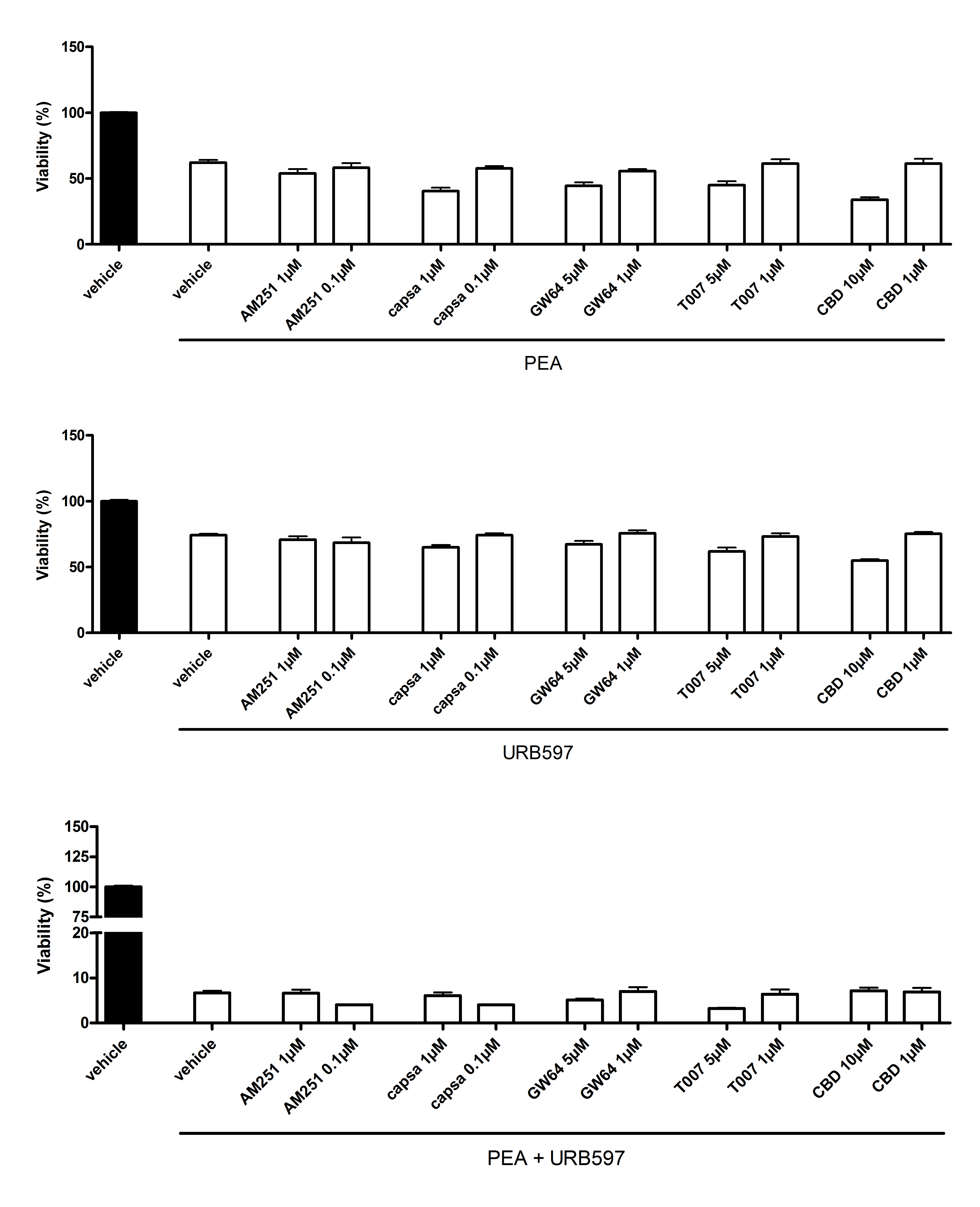

Supplement: Additional file 3 — Investigation of the potential molecular targets of PEA and URB597 in B16 cells. Cytotoxicity of PEA (10 μM), URB597 (10 μM) and PEA + URB597 was not significantly affected by CB1 receptor antagonist (0.1 and 1 μM), TRPV1 receptor antagonist (0.1 and 1 μM), PPAR's receptor antagonists (1 and 5 μM) and GPR55 receptor antagonist (1 and 10 μM). B16 cells were seeded 5 h before treatment (2000 cells/well in microwells) and incubated with PEA alone (10 μM), URB597 alone (10 μM) and combinations of these two molecules. Antagonists were added 1 h prior to the addition of PEA and/or URB597. A MTT test was used to evaluate the percentage of viable cells remaining after 72 h. Data are the mean of three experiments performed in triplicate and are expressed as percentage of the vehicle control. [file 1471-2407-12-92-S3.TIFF]

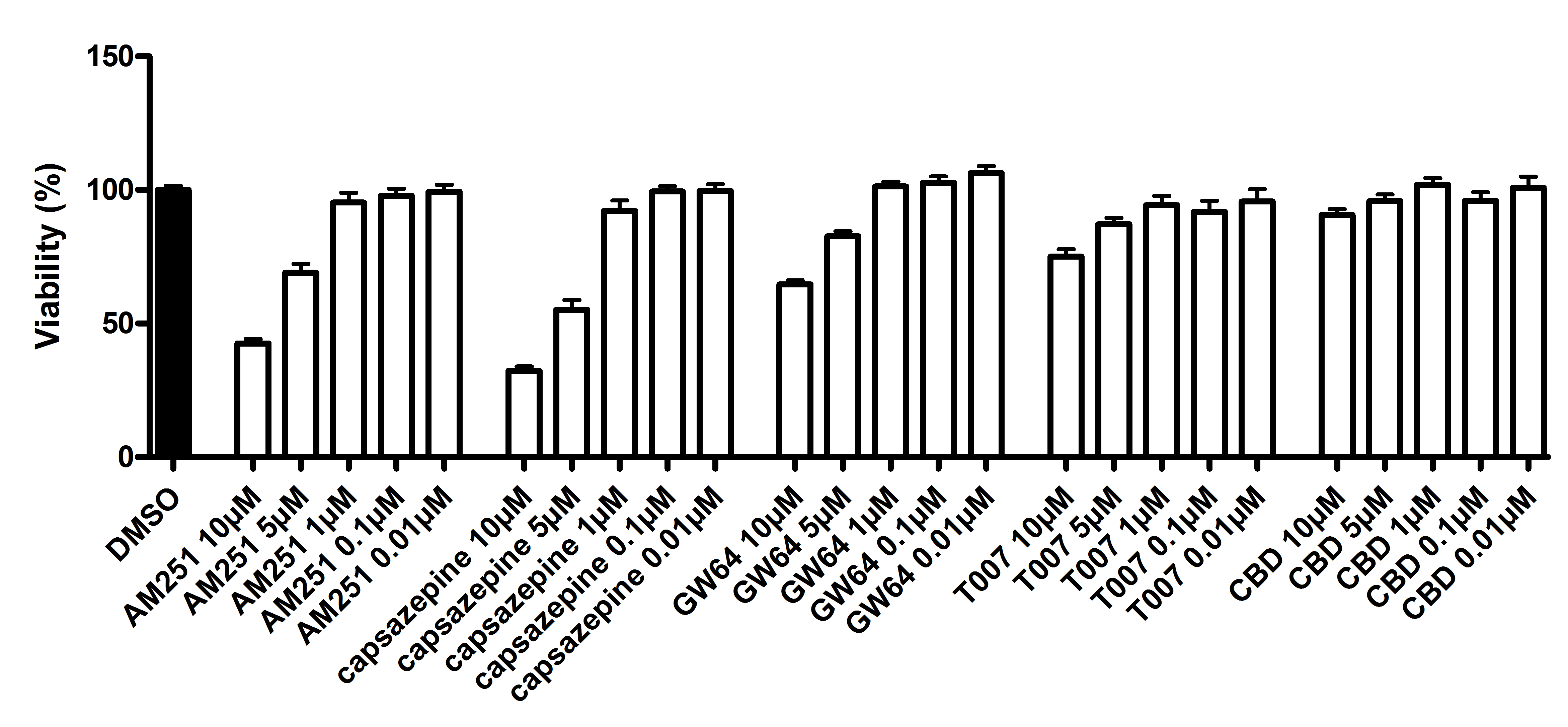

Supplement: Additional file 4 — Cytotoxicity of receptor antagonists. Cytotoxicity of CB1 receptor antagonist (AM251), TRPV1 receptor antagonist (capsazepine), PPARα and PPARγ receptor antagonists (GW6471 and T0070907 respectively) and GPR55 receptor antagonist (cannabidiol, CBD). B16 cells were incubated with the antagonists for 72 h. A MTT test was used to evaluate the percentage of viable cells remaining after treatment. Data are expressed as percentage of the vehicle control and are the mean of three experiments performed in quintuplicate. [file 1471-2407-12-92-S4.TIFF]

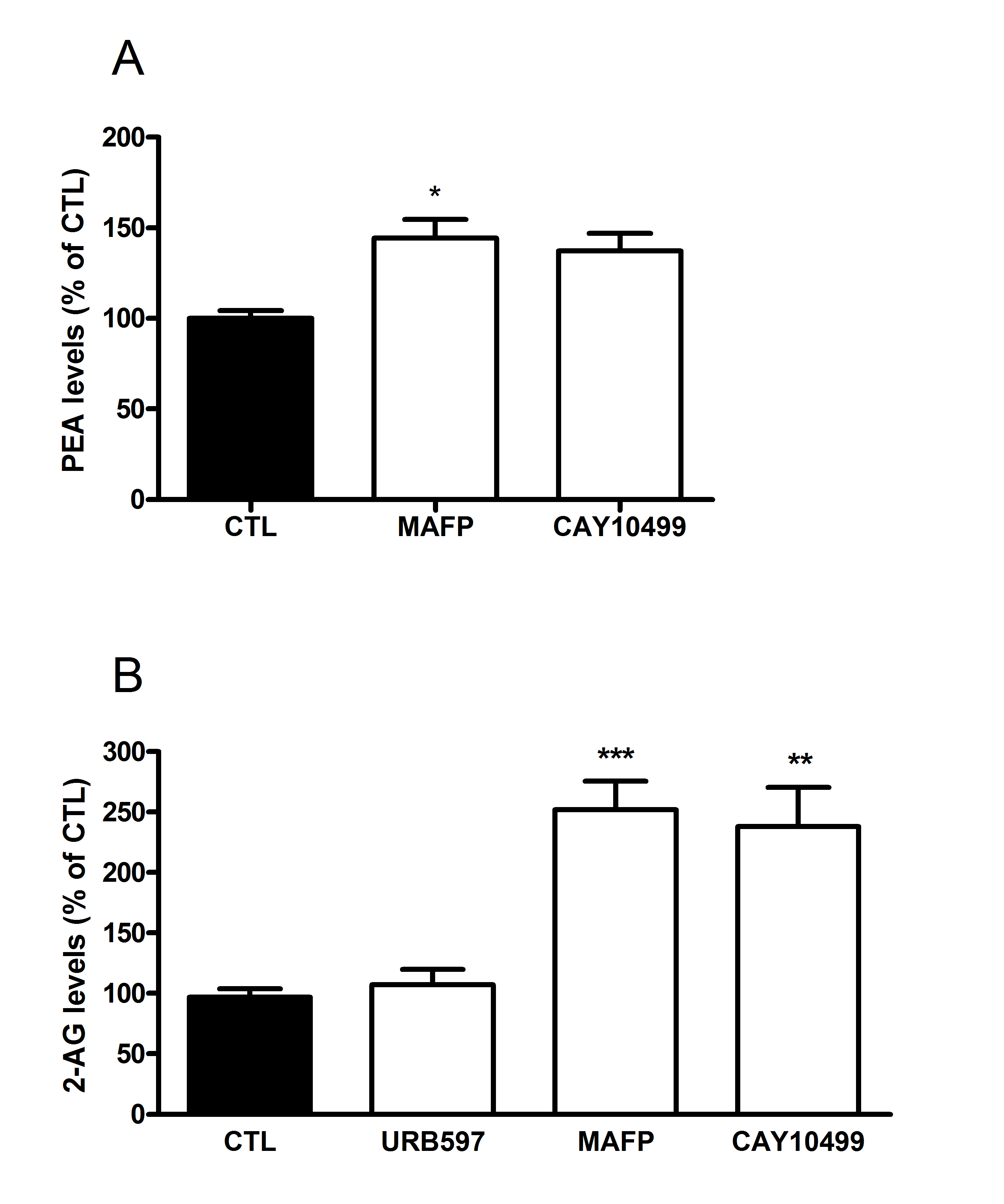

Supplement: Additional file 5 — Effect of MAFP, CAY10499 and URB597 incubation on PEA and 2-AG levels in B16 cells. (A) MAFP, but not CAY10499, increases intracellular levels of PEA. We found in control cells 25.4 ± 3.8 pmol of PEA/107 cells. (B) MAFP and CAY10499, but not URB597, increase intracellular levels of 2-AG. We found in control cells 29.9 ± 4.8 pmol of 2-AG/107 cells. Levels were measured by HPLC-MS. B16 cells (107 cells) were incubated for 8 h with URB597, CAY10499 or MAFP (1 μM). Data are the mean of three experiments performed in quadruplicate and are expressed as percentage of the vehicle control. Significantly different (*P < 0.05; **P < 0.01; ***P < 0.001) from vehicle incubation. [file 1471-2407-12-92-S5.TIFF]
